# Myofiber stress-response in myositis: parallel investigations on patients and experimental animal models of muscle regeneration and systemic inflammation

**DOI:** 10.1186/ar2963

**Published:** 2010-03-24

**Authors:** Maurizio Vitadello, Andrea Doria, Elena Tarricone, Anna Ghirardello, Luisa Gorza

**Affiliations:** 1 Institute of Neuroscience - Padova Section, Consiglio Nazionale delle Ricerche, viale G. Colombo 3, 35121 Padova, Italy; 2 Department of Clinical and Experimental Medicine, Division of Rheumatology, University of Padova, via N. Giustiniani 2, 35128 Padova, Italy; 3 Department of Biomedical Sciences, University of Padova, viale G. Colombo 3, 35121 Padova, Italy

## Abstract

**Introduction:**

The endoplasmic reticulum (ER) stress-response, evoked in mice by the overexpression of class I major histocompatibility complex antigen (MHC-I), was proposed as a major mechanism responsible for skeletal muscle damage and dysfunction in autoimmune myositis. The present study was undertaken to characterize in more detail the ER stress-response occurring in myofibers of patients with inflammatory myopathies, focusing on the expression and distribution of Grp94, calreticulin and Grp75, three ER chaperones involved in immunomodulation.

**Methods:**

Muscle biopsies were obtained from seven healthy subjects and 29 myositis patients, who were subdivided into groups based on the morphological evidence of inflammation and/or sarcolemmal immunoreactivity for MHC-I. Biopsies were analyzed by means of immunohistochemistry and western blot using anti-Grp94, anti-calreticulin and anti-Grp75 specific antibodies. Parallel analyses on these ER chaperones were conducted in rabbit and/or murine skeletal muscle after experimental induction of regeneration or systemic inflammation.

**Results:**

Upregulation of Grp94 characterized regenerating myofibers of myositis patients (*P *= 0.03, compared with values detected in biopsies without signs of muscle regeneration) and developing and regenerating myofibers of mouse muscles. Conversely, levels of calreticulin and Grp75 increased about fourfold and twofold, respectively, in patient biopsies positive for sarcolemmal MHC-I immunoreactivity, compared with healthy subjects and patients negative for both inflammation and MHC-I labeling (*P *< 0.005). Differently from calreticulin, the Grp75 level increased significantly also in patient biopsies that displayed occasional sarcolemmal MHC-I immunoreactivity (*P *= 0.002), suggesting the interference of other mechanisms. Experimental systemic inflammation achieved in mice and rabbits by a single injection of bacterial lipopolysaccharide significantly increased Grp75 and calreticulin but not MHC-I expression in muscles.

**Conclusions:**

These results indicate that, in myositis patients, muscle regeneration and inflammation, in addition to MHC-I upregulation, do evoke an ER stress-response characterized by the increased expression of Grp94 and Grp75, respectively. The increase in the muscle Grp75 level in patients showing occasional immunoreactivity for sarcolemmal MHC-I might be considered further as a broader indicator of idiopathic inflammatory myopathy.

## Introduction

Idiopathic myositis represents a heterogeneous group of chronic autoimmune disorders characterized by an immunomediated inflammatory stress targeted to skeletal muscles [1,2]. Although a large body of evidence supports the role of innate and adaptive immune responses in the pathogenesis of myositis [1,2], the lack of recovery of muscle function observed in patients after immunosuppressive therapies has drawn special interest regarding nonimmune mechanisms of muscle fiber damage [3]. Using transgenic mice, Nagaraju and colleagues showed that the overexpression of class I major histocompatibility complex antigen (MHC-I) in skeletal muscle fibers was responsible for the chronic activation of the endoplasmic reticulum (ER) stress-response and the development of myositis [4]. Although comparable evidence for a causal relationship between MHC-I upregulation and myositis is presently lacking for the human disease, the same authors demonstrated increased transcriptional activity of genes responsive to ER stress, such as the ER chaperone Grp78, in biopsies of myositis patients [4].

Data from the literature suggest that an increased expression of ER chaperones might influence immune mechanisms of fiber damage. ER chaperones favor the assembly of peptide-MHC-I complex, or bind peptides directly [5] - as occurs for Grp94 - and make cells immunogenic after reaching surface localization [6-8] - as described for Grp94, calreticulin and ERp57. Alternatively, chaperones protect against immunological damage; for instance, mtHsp70/Grp75/mortalin [9] protects against complement-mediated cell death through the shedding of the complement C5b-9 membrane attack complex [10].

Except for sporadic inclusion-body myositis, where the ER chaperones calnexin, calreticulin, Grp78, Grp94 and ERp72 are upregulated and colocalize with intracellular aggregates [11,12], the present knowledge about changes in ER chaperone level and distribution among myofibers of myositis patients is far from complete. In particular, data concerning Grp94, calreticulin and Grp75 are presently lacking. A growing body of evidence indicates that Grp94 plays a special role in muscle differentiation and maturation [13-15]. Grp94 is required for mesoderm induction and muscle cell differentiation [14], in so far as it redistributes after Fyn-mediated tyrosine phosphorylation in the secretory pathway [15], where it is probably involved in processing of insulin-like growth factor II [16], and, eventually, localizes at the cell surface, where it participates in myotube formation [13]. Conversely, Grp75 expression in myofibers of human and rat skeletal muscles appears related to oxidative stress occurring either during training of both high and low intensity [17] or during disuse [18].

The aim of the present study was therefore to investigate the stress-response ongoing in muscle fibers of patients affected with inflammatory myopathies, focusing on Grp94, calreticulin and Grp75 expression and distribution. Muscle biopsies obtained from 29 patients were investigated by means of immunohistochemistry and western blot, and results were compared with those obtained from biopsies of healthy subjects. Comparable analyses were then conducted on the experimental animal, in order to determine to which extent conditions that accompany inflammatory myopathies, such as muscle regeneration and systemic inflammation, do contribute to myofiber ER stress-response.

## Materials and methods

### Human studies

Muscle biopsies were collected from seven young male healthy subjects (mean age ± standard error of the mean (SEM), 24 ± 1 years) in compliance with the principles of the Helsinki Declaration [18], and from 29 patients positive for at least two of Bohan and Peter's criteria for inflammatory myopathy [19]. Data were stripped of personally identifiable information. The study was approved by the local ethics committee (University of Padova - Azienda Ospedaliera di Padova, Italy) and written informed consent for study participation was obtained from all the healthy subjects and from patients.

Data concerning sex, disease duration, creatine phosphokinase levels in serum, the presence of muscle weakness and of dermatomyositis skin rash, and the positivity for electromyographic signs of myogenic disease are presented in Table 1. Muscle weakness was defined according to Bohan and Peter criteria [19], as symmetrical weakness of the limb-girdle muscles and anterior neck flexor progressing over weeks to months, reported by patients and confirmed by clinical evaluation of muscle strength. The presence of antinuclear autoantibodies was routinely determined by indirect immunofluorescence on HEp2 cells and by immunoblotting, as previously described [20,21]. A positive antinuclear autoantibody test was defined as serum titer ≥ 160. Myositis-specific autoantibodies, including anti-Mi-2, anti-t-RNA synthetases and anti-signal recognition particle, were determined by a modified immunoblot assay and indirect immunoprecipitation of cognate RNAs, respectively [22]. Briefly, IgG antibodies to Mi-2 antigen were detected by chemiluminescent immunoblot on 7% SDS-PAGE-resolved nuclear extract from Raji cells. Antibodies to RNA moieties were identified by protein A-sepharose-assisted immunoprecipitation from patient sera incubated with Jurkat cell lysate and analysis by silver staining of 7% polyacrylamide/urea gels.

**Table 1:** Clinical, biochemical, immunological and immunohistological features of myositis patients

Patient	Sex	Disease duration (months)	Muscle weakness	DM skin rash	Serum CPK^a ^(U/l)	EMG^b^	ANA (IF)^b^	Specific autoantibodies	Muscle biopsy^c^		
									
									Inflammation (site of biopsy)	Immunoreactivity for	
										
										MHC-I	εMy (% of total)
Group I											
1	F	4	P	A	5027	+	-	Jo-1, RoSSA	++ (VL)	++	36.60
2	F	15	P	A	8570	+	-	Jo-1	++ (D)	++	35.50
3	F	8	P	A	2762	-	+	Jo-1, RoSSA	++ (VL)	+	15.50
4	F	168	P	A	1100	+	-	-	++ (VL)	++	1.09
5	M	29	P	A	1125	-	+	Mi-2	++ (VL)	+	0.33
6	F	13	P	P	292	+	-	-	++ (VL)	++	0
7	F	3	P	A	6705	+	-	Jo-1	+ (VL)	+	7.79
8	F	25	A	A	1840	+	+	tRNA, RoSSA	+ (VL)	++	2.00
9	M	25	P	P	484	+	-	Mi-2	+ (D)	++	6.00
10	M	79	P	A	535	-	+	U1RNP, RoSSA	+ (VL)	++	36.60
11	F	9	P	A	2762	+	+	-	+ (VL)	+	15.55
12	F	13	P	A	666	+	-	-	+ (TA)	++	3.10
13	M	36	P	A	2580	-	+	tRNA	+ (VL)	+	0.20
14	F	103	P	A	893	+	+	Jo-1	+ (VL)	+	0.06
Group II											
15	F	27	P	A	173	-	+	Jo-1	- (VL)	+	0.13
16	M	72	P	A	548	-	-	-	- (VL)	+	0
17	F	79	P	A	< 160	-	+	Ro/SSA	- (D)	++	0
18	M	33	P	P	600	ND	+	-	- (D)	++	0.42
19	F	21	P	A	221	+	+	Scl70, Ku	- (VL)	++	2.60
20	F	180	P	A	1157	+	+	Jo-1	- (VL)	+	0
21	F	67	P	A	533	+	+	Mitotic apparatus	- (D)	+	1.1
Group III											
22	F	237	P	A	450	-	-	-	- (VL)	-	0.50
23	F	58	P	A	43	+	-	-	- (VL)	-	0
24	F	237	P	A	742	+	-	-	- (D)	-	0
25	M	24	P	A	250	-	-	-	- (VL)	-	0
26	F	15	P	A	1600	-	-	-	- (VL)	-	1.11
27	F	68	P	A	628	+	-	-	- (VL)	-	0
28	M	33	P	P	9868	+	-	-	- (VL)	-	0
29	F	24	P	P	254	-	+	-	- (VL)	-	0

^a^Values correspond to those obtained at the time of muscle biopsy. ^b^+ and -, presence and absence of positive response, respectively. ^c^+ and ++, degree of inflammation and MHC-I immunofluorescence, evaluated as described in Materials and methods; -, absence. DM, dermatomyositis; CPK, creatine phosphokinase; EMG, electromyography; ANA (IF), antinuclear antibodies (immunofluorescence); MHC-I: class I major histocompatibility complex antigen; εMy (% of total), percentage of myofibers positive for embryonic skeletal myosin on total biopsy fibers; F, female; M, male; P, present; A, absent; VL, vastus lateralis muscle; D, deltoid muscle; TA, tibialis anterior muscle; ND, not done.

Muscle biopsy was obtained after local anesthesia, according to standard techniques [18]. The vastus lateralis muscle was biopsied in volunteers and in the large majority of patients (Table 1). From each biopsy, a sample of about 20 to 30 mg was removed, frozen in liquid nitrogen and stored at -80°C for the present study. In the case of patients, the remaining tissue was processed for routine histology.

### Animal experimental studies

Thirteen 1-day-old embryos and adult (30 g) male CD-1 mice were used. Samples from the liver, heart and tibialis anterior muscle were excised from adult animals. Samples were frozen in liquid nitrogen and stored at -80°C. Eight adult mice were injected with 30 to 50 μl of 50 mM BaCl_2 _in the tibialis anterior to induce muscle degeneration and subsequent regeneration [23]. Animals were sacrificed at days 3, 4 and 15 after surgery. For western blot analysis, the superficial inflamed region of the tibialis anterior was excised from the bulk of the muscle; whereas for immunohistochemistry, the whole muscle was taken.

To improve morphological preservation of the regenerating area, some mice were perfused under anesthesia with PBS (NaCl 136 mM, KCl 2.68 mM, Na_2_HPO_4 _8 mM, KH_2_PO_4 _1.4 mM, pH 7.4), followed by 4% paraformaldehyde in PBS. Samples were then excised, incubated in fixative for a further 2 hours at 4°C, rinsed with PBS, cryoprotected with increasing concentrations of sucrose and frozen.

Adult mice (n = 6) were treated with a single intraperitoneal injection of lipopolysaccharide (LPS) from *Salmonella typhimurium *(1 mg/kg; Sigma, Milan, Italy); control animals (n = 6) were injected with saline. Animals were euthanized 48 hours after the injection, were weighed and the tibialis anterior muscles were excised and frozen. The LPS treatment of New Zealand rabbits was described previously [24].

Five-week-old male mice from the C57BL/10 strain (n = 5) and the dystrophin mutant *mdx *strain (n = 4) were sacrificed, and the tibialis anterior and gastrocnemius muscles were excised and frozen.

Each experimental protocol followed internationally recognized guidelines and was approved by the Animal Care Committees of the University of Padova and the Italian Ministry of Public Health.

### Immunofluorescence and immunohistochemical analyses

Serial consecutive 12 μm cryosections were prepared from frozen muscle samples, collected on gelatin-coated glasses, and assayed for immunofluorescence and indirect peroxidase immunohistochemistry, following previously described protocols.

Immunofluorescence labeling was used with fluorescein isothiocyanate (FITC)-labeled anti-human HLA-ABC antibody (clone W6/32; Serotec, Oxford, UK). Sections were dried for 40 minutes, fixed for 15 minutes with acetone at -20°C and dried. Cryosections were then incubated at room temperature with 1:50 dilution of the anti-HLA antibody, rinsed with PBS and mounted with glycerol containing 0.01% 4,6-diamidino-2-phenylindole dihydrochloride (DAPI) to counterstain nuclei. The presence of positive staining for MHC-I was evaluated with a microscope equipped with fluorescence optics (Axioplan; Carl Zeiss Italia, Milan, Italy) and graded, according to van der Pas and colleagues [25], as follows: grade -, undetectable in myofibers, but present on capillaries; grade +, both capillaries and myofiber sarcolemma are stained, but the capillaries can still be identified easily; and grade ++, both capillaries and myofiber sarcolemma are stained, but capillaries can no longer be identified. MHC-I immunostaining was further enhanced by incubating sections for 30 minutes at 37°C with a 1:40 dilution of mouse anti-FITC immunoglobulins conjugated with peroxidase (Roche Diagnostic, Milan, Italy) and developed as described below. Immunoreactivity for murine MHC-I was visualized by incubating sections with a 1:5 dilution of a monomorphic mouse monoclonal antibody labeled with FITC (MCA2189F; Serotec), as described above, and by subsequent amplification of antibody binding using the anti-FITC antibody conjugated with peroxidase.

For indirect immunoperoxidase staining, sections were processed as previously described [18,24,26]. Fiber typing was achieved by comparison of serial sections labeled with the monoclonal anti-slow β-myosin heavy chain (My) antibody BA-D5 [18], which identifies type 1 fibers, with those stained for the succinate dehydrogenase, which discriminates between red and white myofibers [27]. Regenerating myofibers were identified by reactivity with the monoclonal anti-My antibody BF-G6, which recognizes the human embryonic My isoform (εMy) [28]. Stress-protein expression was monitored with the following antibodies: anti-Grp75 mouse monoclonal antibody (SPS-825; Stressgen, Victoria, BC, Canada) [18]; anti-Grp94 mouse monoclonal antibody (clone 3C4) [24,26] and rabbit polyclonal antibody (SPA-851; Stressgen); anti-calreticulin rabbit polyclonal antibodies (SPA-600; Stressgen); and anti-CHOP/GADD153 rabbit polyclonal antibodies (R-20; Santa Cruz Biotech, Heidelberg, Germany). The presence of necrosis and autophagy was determined using mouse monoclonal antibody for human complement C9 (Novocastra Laboratories, Newcastle upon Tyne, UK) and anti-ubiquitin rabbit polyclonal antibody (DakoCytomation, Milan, Italy), respectively, as described by Kostin and colleagues [29].

Specificity of the staining was checked by processing adjacent sections with the same protocol, except for the use of nonimmune mouse or rabbit immunoglobulins (1 μg/ml; Sigma) as the primary antibody.

Consistency of the immunostaining was validated by independent analysis.

### Western blotting

Procedures for western blotting were previously described [18]. Equal amounts of muscle proteins were tested with the following antibodies: anti-generic My, mouse clone BF-46 [18]; anti-Grp94 3C4 [25]; anti-Grp75 SPS-825 [18]; anti-calreticulin SPA-600; and anti-α-actinin, mouse clone EA-53 (Sigma). Quantitative densitometry was performed analyzing autoradiographic bands using Image J software (NIH Bethesda, MD, USA). Values were normalized to the corresponding densitometric value of the Ponceau red staining of the actin band [18] or of the α-actinin immunostaining.

### Statistical analysis

All data are expressed as the mean ± SEM. Statistical analysis was performed utilizing unpaired analysis (one-way analysis of variance (ANOVA) and *post-hoc t*-test). *P *= 0.05 was set as the limit for significance. All these analyses and linear regression analyses were performed using the SigmaStat version 2.0 statistical package (Jandel, Germany).

## Results

### Human studies

#### Characterization of the patient cohort

All patients referred with longstanding muscle weakness and showed increased levels of serum creatine phosphokinase, although positive electromyography for myogenic suffering and positive histology for inflammation were present in less than one-half of them (Table 1). Diagnosis of polymyositis or dermatomyositis was based on Bohan and Peter's criteria [19] (muscle weakness, increased serum creatine phosphokinase levels, electromyography myopathic signs, positive histology, for the former one, plus the presence of specific skin rash, for the latter one). Connective tissue disease overlap syndrome was diagnosed in Patients 16, 17 and 19. Patients 23 and 25 were included in the study because of the presence of two items from Bohan and Peter's criteria even though the diagnosis was myogenic suffering of unknown origin. None of the patients was affected with sporadic inclusion body myositis or genetic inflammatory myopathy.

Patients were divided to three groups based on the presence of inflammation at routine histology and MHC-I immunofluorescence (Table 1). Group I included patients with either intense (grade ++) or weak (grade +) signs of inflammation and the presence of sarcolemmal immunoreactivity for MHC-I, graded as described in Materials and methods. Group II included patients without histological signs of inflammation and with sarcolemmal MHC-I immunoreactivity. Group III included patients without both signs of inflammation and immunoreactivity for MHC-I in muscle fibers. Prolonged and intense immunosuppressive treatment before biopsy was excluded (Additional file 1). Both sexes were represented in patient groups, with a predominance of females. No statistically significant difference was detected in average age among patient groups (mean age ± SEM: 56.8 ± 4.2 years for Group I, 58.7 ± 6.9 years for Group II, and 57.8 ± 4.6 years for Group III).

The majority of patients enrolled in Groups I and II showed the presence of myositis-specific antibodies; that is, anti-tRNA-synthetases (anti-Jo-1 antibody) and -Mi-2 antigen, and/or myositis-associated autoantibodies (Table 1). Conversely, none of these antibodies was detected among patients of Group III.

The presence of muscle regeneration was evaluated on biopsies by counting εMy-positive myofibers [28] and expressing the value as the percentage of the total fiber number (Table 1). There were more than 500 fibers in the large majority of biopsies. The degree of regeneration was higher in Group I patients, although it was not always consistent with the histological evidence of inflammation (Table 1). Regeneration was negligible in patients of Groups II and III.

**Figure 1:**
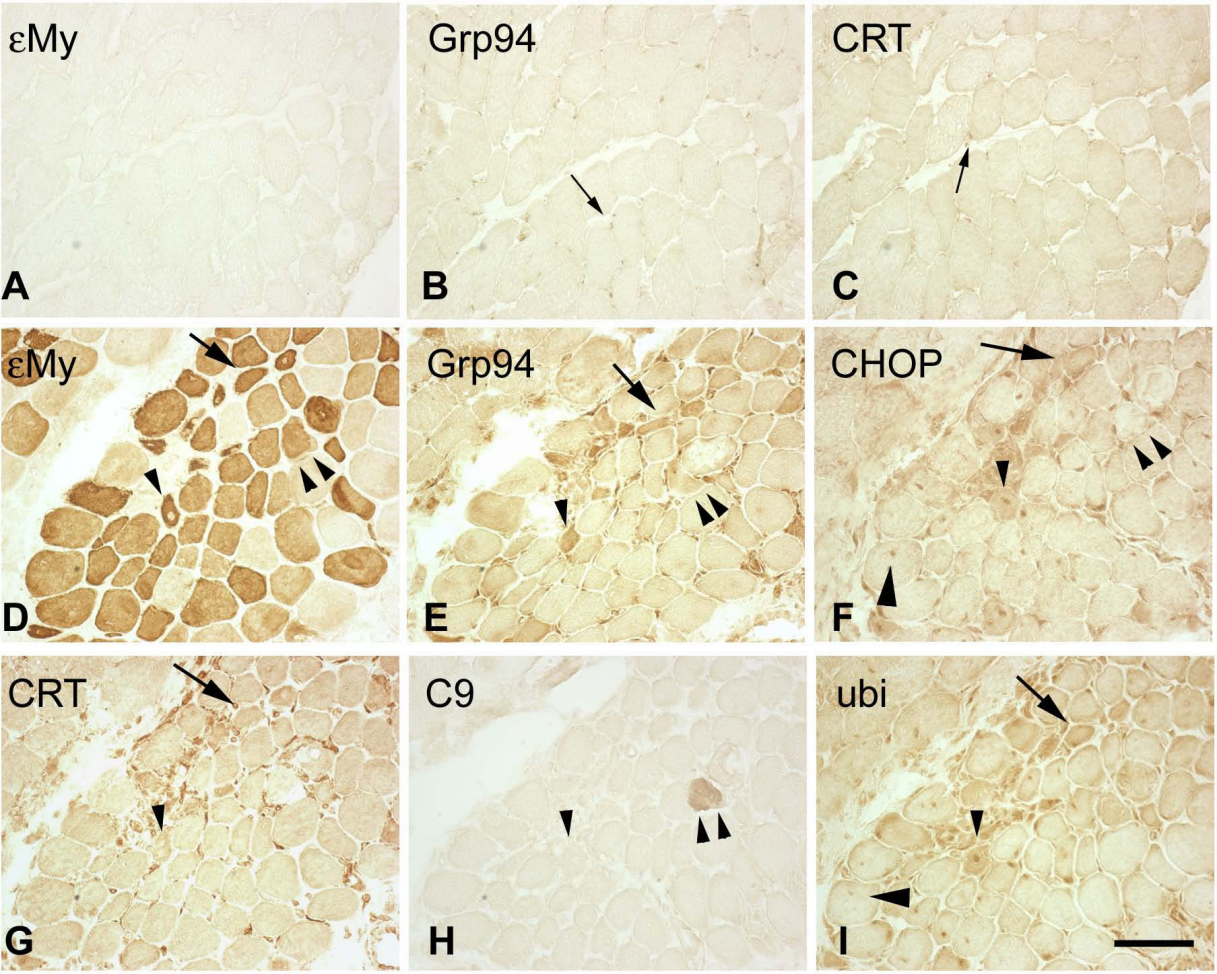
**Immunoreactivity for endoplasmic reticulum stress-markers in regenerating skeletal myofibers of myositis patients**. Serial cryosections from biopsies of **(a) **to **(c) **a Group 0 subject (S6) and **(d) **to **(i) **a Group I myositis patient (P2), who displayed a high degree of muscle regeneration, were stained with indirect immunoperoxidase with antibodies for embryonic skeletal myosin heavy chain (εMy), Grp94, calreticulin (CRT), CHOP, complement 9 (C9) and ubiquitin (ubi). Thin arrows in (b) and (c) indicate interstitial cells reacting with anti-Grp94 and anti-caltreticulin antibodies. Thick arrows in (d) to (i) indicate a regenerating muscle fiber (εMy-positive), which stained positively for Grp94 (e), cytosolic CHOP (f), calreticulin (g) and ubiquitin (f). Thick arrowheads in (f) and (i) indicate a regenerating muscle fiber, which displayed nuclear CHOP (f) and ubiquitin (i) immunoreactivity. Arrowheads in (d) to (i) indicate a regenerating muscle fiber, which displayed positive immunoreactivity for Grp94 (e) and calreticulin (g), and both cytosolic and nuclear CHOP (f) and ubiquitin (i) staining. Double arrowheads in (d) to (h) indicate the presence of positive C9 immunoreactivity, a marker of necrosis (h), in a regenerating muscle fiber, which displayed positive immunoreactivity for Grp94 (e). Bar: 100 μm.

Biopsies from healthy volunteers corresponded to Group 0 and were used as reference samples. No immunoreactivity for MHC-I was detected in muscle fibers (not shown) and no muscle regeneration was observed (Figure 1a).

### Immunohistochemical analyses

#### Healthy subject biopsies (Group 0)

**Figure 2:**
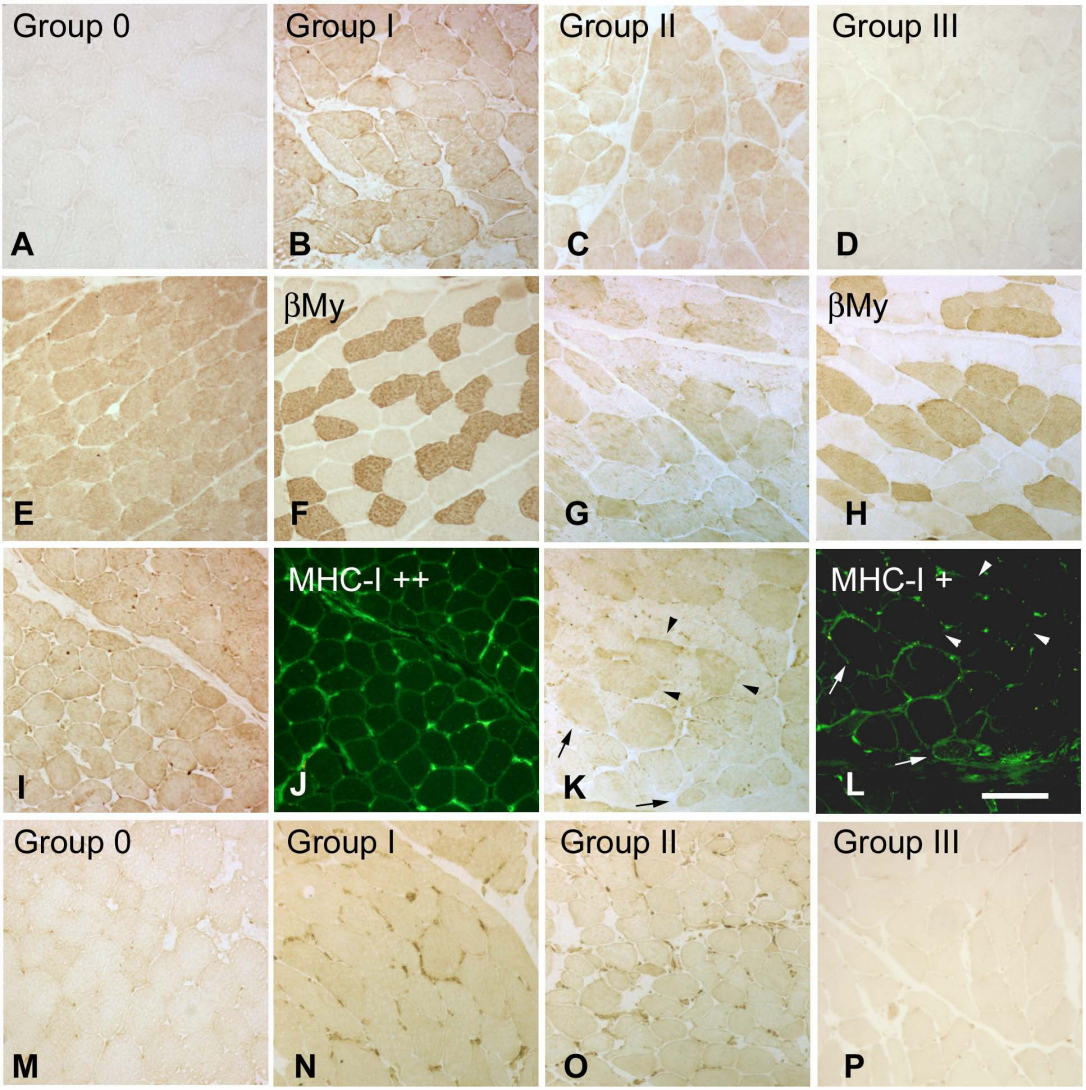
**Distribution of Grp75 and calreticulin immunoreactivity in adult skeletal myofibers of myositis patients**. Representative immunoreactivity for Grp75 in cryosections from biopsies of **(a) **a Group 0 subject (S7) and of myositis patients (**(b) **P5 from Group I, **(c) **P20 from Group II, and **(d) **P22 from Group III) after immunoperoxidase staining. **(e) **to **(h) **Distribution of Grp75 immunoreactivity with indirect immunoperoxidase in two additional Group I patients ((e) P7, and (g) P14) and that of type 1 fibers, identified by the immunoperoxidase staining for β-myosin heavy chain (βMy) of adjacent cryosections (f) and (h). **(i) **to **(l) **Comparison of the distribution of Grp75 immunoperoxidase labeling of two Group I patients, who differed for the degree of class I major histocompatibility complex antigen (MHC-I) immunoreactivity ((i) P9, grade ++ MHC-I; (k) P14, grade + MHC-I) with the presence of sarcolemmal immunofluorescence for MHC-I (j) and (l). Thin arrows in (k) and (l) indicate Grp75-positive and sarcolemmal MHC-I-positive fibers, whereas arrowheads indicate the presence of Grp75 immunoreactivity in sarcolemmal MHC-I-negative ones. **(m) **to **(p) **Representative histochemical demonstration for calreticulin immunoperoxidase in cryosections from biopsies of (m) a Group 0 subject (S1) and of myositis patients ((m) P6 from Group I, (o) P19 from Group II, and (p) P22 from Group III). Bar: 100 μm.

Immunohistochemistry for ER chaperones showed negligible reactivity for Grp94 and Grp75 in muscle fibers of biopsies from Group 0 subjects (Figures 1b and 2a for Grp75), whereas antibodies against calreticulin labeled weakly a number of muscle fibers (Figures 1c and 2m), which corresponded to about 60% of the type 1 population and to 10% of the type 2 population one (data not shown). Interstitial cells and vascular endothelial cells were labeled by Grp94 and calreticulin antibodies (arrows in Figure 1b, d). A faint cytosolic labeling for the ER-stress-induced transcription factor CHOP [30] was detected in type 2 fibers (Additional file 2A). Signs of muscle death were apparently absent from biopsies of Group 0 subjects (Additional file 2B,C). No fiber showed positive immunoreactivity for ubiquitin, a marker of proteasome overload and autophagic death [29], and for serum complement protein C9, whose intracellular localization is indicative for the presence of necrosis [29].

#### Patient biopsies with a high percentage of regenerating myofibers (Group I)

Different patterns of immunohistochemical labeling for Grp94, Grp75 and calreticulin were detected in biopsies from the patient groups (Figures 1 and 2). Figure 1d to 1i represents the staining observed in those Group I patients who displayed a high percentage of regenerating myofibers. Labeling for εMy was observed in variably sized fibers: small reactive myofibers with centrally located nuclei (arrowhead) corresponded to newly formed myotubes, whereas larger myofibers corresponded to more advanced maturation stages (Figure 1d). The majority of εMy-positive myofibers showed positive immunoreactivity for Grp94 (Figure 1e). A comparable picture was observed for calreticulin immunostaining, although the presence of labeling in adult myofibers made less appreciable the decoration of regenerating myofibers (Figure 1g). Some of the Grp94-positive and calreticulin-positive myotubes also displayed strong labeling for CHOP [30], whose staining localized either in the nucleus (thick arrowhead), in the cytosol (thick arrow), or at both locations (single arrowhead) (Figure 1f; compare with Additional file 2A). A minor proportion of CHOP-positive myotubes also showed positive nuclear and cytosolic immunostaining for ubiquitin (Figure 1i, thick arrows and thick arrowheads, respectively). The presence of intracellular positive staining for C9 was occasionally observed among either CHOP-negative (Figure 1h, double arrowhead) or CHOP-positive regenerating myofibers (data not shown). Regenerating myofibers were not apparently labeled by anti-Grp75 antibodies (data not shown; see Additional file 3).

This same pattern of ER stress-protein immunoreactivity was also observed in regenerating myofibers in the presence of nonprimary inflammatory myopathy, such as Duchenne dystrophy [31] (see Additional file 3A to 3E).

#### Patient biopsies with a low percentage or absence of regenerating myofibers (Groups I to III)

Nonregenerating myofibers of myositis patients differed in immunoreactivity for Grp75 and calreticulin (Figure 2), although none of the patients studied showed an immunoreactivity pattern compatible with intracellular aggregates.

Staining for Grp75 appeared more intense in myofibers of Group I and II patients, compared with those of Groups 0 and III (Figure 2a to 2d). The increased reactivity was detectable in the large majority of muscle fibers, always encompassing type 1 and type 2A fiber populations (Figure 2e to 2h), and appeared homogeneously distributed within the cytosol. Furthermore, immunolabeling for Grp75 was not obligatorily related to the presence of sarcolemmal MHC-I immunoreactivity, in so far as it was detected also in those Group I and II patients who displayed MHC-I immunofluorescence only in a minor proportion of muscle fibers (grade +) (Figure 2k, l - arrows indicate MHC-I-positive fibers, arrowheads indicate negative fibers; compare with Figure 2i, j). Grp75 immunostaining was also absent from nonregenerating myofibers of nonprimary inflammatory myopathy, such as Duchenne dystrophy (Additional file 3A to 3E).

Calreticulin immunoreactivity also appeared to distribute to a larger fiber population in biopsies of Group I and Group II myositis patients (Figure 2m to 2o) than that one of healthy subjects, whereas staining of biopsies of Group III patients (Figure 2p) was comparable with that of Group 0 subjects. We then investigated whether a change in fiber type composition of patients of Group I and Group II might explain the wider distribution of calreticulin. The percentage of type 2A fibers significantly decreased in both groups, compared with Group 0 (mean ± SEM: 23.94 ± 3.91%, 18.52 ± 3.90%, and 38.44 ± 3.95% for Group I, Group II, and Group 0, respectively; n = 5, *P *= 0.01, ANOVA), whereas the relative percentage of type 1 fibers increased by about 30%, albeit above the limit set for statistical significance (mean ± SEM: 42.59 ± 4.30%, 46.89 ± 3.89%, and 33.56 ± 3.93% for Group I, Group II, and Group 0, respectively; n = 5, *P *= 0.06, ANOVA). Nevertheless, this moderate increase in the type 1 fiber population did not quite explain the wider distribution of calreticulin immunoreactivity in biopsies of Group I and Group II patients.

Nonregenerating myofibers of Group I patients showed occasional immunoreactivity for CHOP (Additional file 4). Reactive fibers displayed also intense staining for both calreticulin and Grp94 (data not shown) and were often positive for C9 (arrows in Additional file 4) and ubiquitin (data not shown). Except for these necrotic myofibers, immunostaining for Grp94 appeared negligible in nonregenerating myofibers (data not shown). Granular Grp94 immunoreactivity compatible with the presence of intracellular aggregates was never observed.

### Western blot analyses

**Figure 3:**
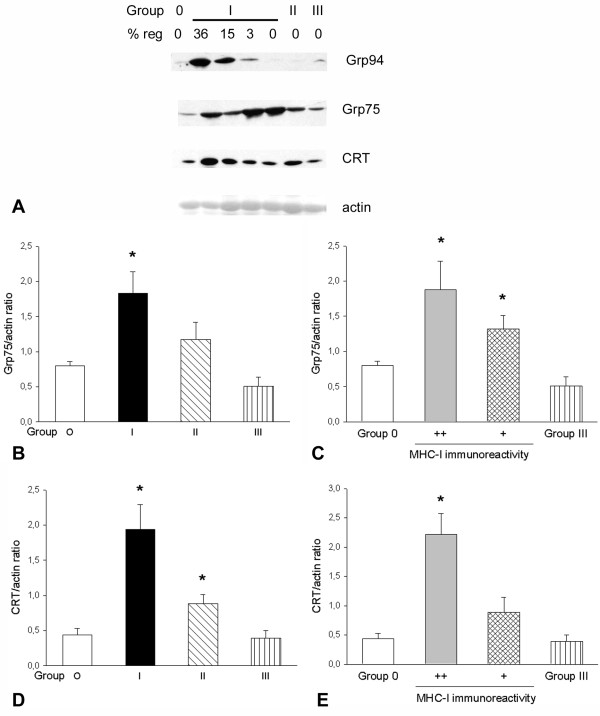
**Expression levels of Grp94, Grp75 and calreticulin in patients' biopsies**. **(a) **Representative western blots of biopsies from Group 0 (S3), Group I (P1, P2, P12, P13), Group II (P20), and Group III patients (P25) stained for Grp94, Grp75, calreticulin (CRT) and actin. The percentage of regenerating fibers (% reg) is indicated above each patient lane. **(b) **Histograms showing mean and standard error of the mean (SEM) for the normalized Grp75 amount detected in Groups 0, I, II and III (n = 7, 14, 7 and 8, respectively). *Significant difference versus Groups 0 and III (*P *= 0.01, analysis of variance (ANOVA)). **(c) **Histograms show mean and SEM of normalized Grp75 amount compared after redistribution of Group I and Group II patients into two new groups based on grading of class I major histocompatibility complex antigen (MHC-I) sarcolemmal immunostaining (see Table 1; n = 11 for MHC-I grade ++ group, 10 for MHC-I grade + group). *Significant difference versus Groups 0 and III (*P *< 0.005, ANOVA). **(d) **Histograms showing mean and SEM of normalized calreticulin amount detected in Groups 0, I, II and III (n = 6, 14, 7 and 7, respectively). *Significant difference versus Groups 0 and III (*P *< 0.01, ANOVA). **(e) **Histograms showing mean and SEM of normalized calreticulin amount compared after redistribution of Group I and Group II patients, as described for (c). *Significant difference versus all of the groups (*P *< 0.01, ANOVA).

The differences in ER stress-protein staining observed in immunohistochemistry were validated by western blot analysis (Figure 3).

#### Grp94

Increased signals for Grp94 were observed in those patients of Group I who displayed the presence of regenerating myofibers (mean ± SEM Grp94/actin ratio, 3.23 ± 1.27; n = 5), compared with values observed in biopsies from Groups 0, I and II, in the absence of muscle regeneration (mean ± SEM Grp94/actin ratio, 0.43 ± 0.05; n = 6, *P *= 0.03).

#### Grp75

The relative amount of Grp75 significantly increased twofold in Group I patients, compared with Group 0 and Group III levels (Figure 3b; *P *= 0.01, ANOVA). Interestingly, the level of statistical significance increased when the comparison was performed considering only the Group I biopsies, which displayed the lowest degree of muscle regeneration (mean ± SEM Grp75/actin ratio of this subgroup, 2.26 ** ± **0.56; n = 7; *P *< 0.005 compared with Group 0 values). Conversely, Grp75 level detected in Group II patients did not differ statistically from that observed in Group 0 subjects, whereas it appeared significantly higher than that of Group III patients (*P *= 0.02), whose average Grp75 level was lower than that of Group 0 (*P *= 0.056) (Figure 3b). Statistical evaluation of normalized Grp75 protein levels was then performed after redistribution of Group I and Group II patients in subgroups based on the grading of MHC-I immunoreactivity. Figure 3c shows that biopsies from patients with a sarcolemmal MHC-I expression detectable only in a proportion of myofibers (grade +; n = 10) displayed significantly increased Grp75 levels compared with values detected in Group 0 and Group III subjects (*P *= 0.002, ANOVA), similarly to those detected in biopsies with ubiquitous distribution of sarcolemmal MHC-I immunoreactivity (grade ++).

#### Calreticulin

The relative amount of calreticulin significantly increased about fourfold and twofold in patients of Groups I and II, respectively, compared with the protein levels detected in Group 0 and Group III biopsies (*P *= 0.003, ANOVA) (Figure 3a, d). When normalized calreticulin protein levels were evaluated after redistributing Group I and Group II patients in subgroups based on the degree of MHC-I immunoreactivity, only the biopsies from patients with sarcolemmal MHC-I reactivity detectable in every myofiber (grade ++) displayed a significant increase in calreticulin levels, compared with the levels detected in the other groups (*P *< 0.001, ANOVA) (Figure 3e).

### Animal experimental studies

In order to ascertain whether the differential upregulation of these ER chaperones in regenerating and adult myofibers of myositis patients reflected disease-related mechanisms, their expression was investigated in experimental animal models of muscle regeneration and systemic inflammation.

#### Muscle regeneration

**Figure 4:**
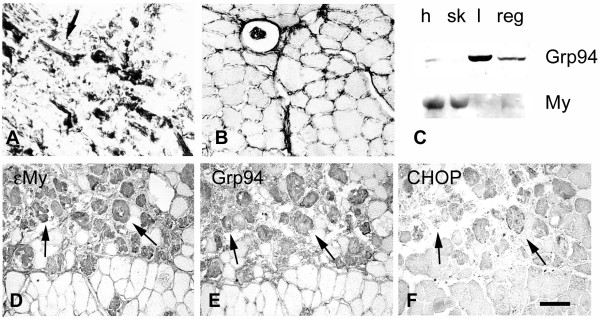
**Grp94 and CHOP protein expression in regenerating murine myofibers**. **(a)**, **(b) **Indirect immunoperoxidase staining of murine cryosections with the anti-Grp94 antibody. (a) Immature skeletal muscle from a 13-day mouse embryo; embryonic myofibers appear darkly stained and the arrow indicates the presence of a sarcomeric pattern of labeling. (b) Transverse section of adult tibialis anterior muscle; myofibers appear unlabeled; the reticular immunostaining around myofibers is due to the reactivity of the secondary antibody with mouse immunoglobulins in the interstitial space. **(c) **Western blotting analysis with Grp94 of whole homogenates prepared from adult mouse myocardium (h), tibialis anterior muscle (sk), liver (l) and the superficial region of a tibialis anterior muscle, 4 days after injury to induce muscle regeneration (reg). Labeling of the myosin heavy chain (My) is shown as a reference for loading. **(d) **to **(f) **Indirect immunoperoxidase staining of cryosections from adult tibialis muscle, 4 days after induction of muscle regeneration, with (d) monoclonal mouse anti-embryonic skeletal myosin, (e) anti-Grp94 antibodies and (f) polyclonal rabbit anti-CHOP antibodies. Arrows in (d) to (f) indicate regenerating myofibers with centrally located nuclei. Bar: 50 μm.

Because muscle regeneration recapitulates muscle differentiation [32] and Grp94 showed a developmentally regulated expression in rabbit skeletal muscle [24], we investigated murine developing muscle by immunohistochemical and western blot analyses. Cryosections from a 13-day embryo and from adult tibialis anterior muscle showed positive staining for Grp94 in immature myofibers (Figure 4a, arrow), whereas labeling was absent from adult myofibers (Figure 4b). Western blot analysis showed a single reactive polypeptide of apparent molecular weight 99,000 in adult myocardium and liver samples; no reactivity was detected with adult skeletal muscle homogenate (Figure 4c, lanes h, sk and l).

Muscle regeneration was then induced in the tibialis anterior of adult mice [23]. Western blot analyses of homogenates prepared from the superficial region of the muscle after 4 days of regeneration showed the labeling of a polypeptide with the same electrophoretic mobility of Grp94 (Figure 4c, lane reg). Immunohistochemistry showed the presence of an abundant number of regenerating muscle cells and myotubes, identified by their reactivity for εMy (Figure 4d). Regenerating myofibers reacted strongly for Grp94 (Figure 4e), whereas they showed variable immunoreactivity for CHOP (Figure 4f, arrows) and calreticulin (data not shown). Positive staining for ubiquitin was occasionally observed (data not shown).

Muscle regeneration was accompanied by increased expression of MHC-I, as shown by the presence of cytosolic immunoreactivity in regenerating myofibers (Additional file 3F to 3G), consistent with that described in the human [33].

#### Systemic inflammation

**Figure 5:**
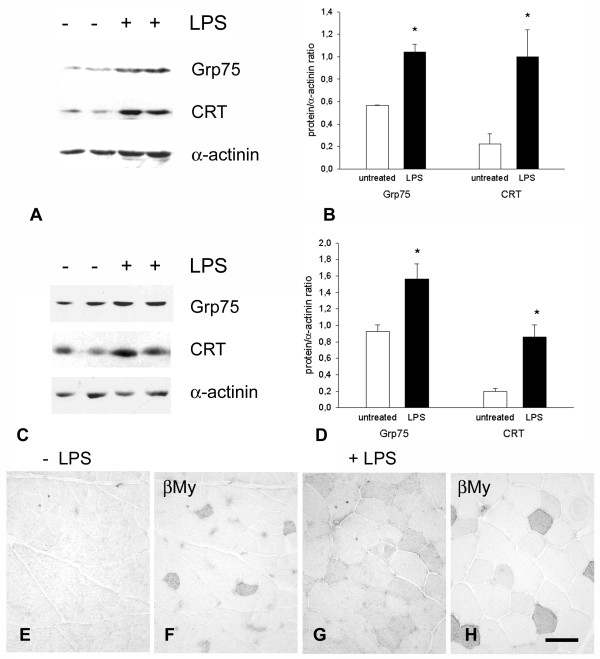
**Skeletal muscle expression of Grp75 and calreticulin after lipopolysaccharide injection in mice and rabbits**. **(a) **Representative western blots of tibialis anterior muscle of adult mice, left untreated or 48 hours after a single injection of lipopolysaccharide (LPS), labeled with antibodies for Grp75 and calreticulin (CRT). Staining of α-actinin is shown as a reference for sample loading. **(b) **Histograms showing mean and standard error of the mean (SEM) for the normalized amount of muscle Grp75 levels of untreated and LPS-treated mice. *Significant difference versus untreated muscles (*P *< 0.01). **(c) **Representative western blots of hindlimb muscles of adult rabbits, left untreated or 48 hours after a single injection of LPS, labeled with antibodies for Grp75 and CRT. Staining of α-actinin is shown as a reference for loading. **(d) **Histograms showing mean and SEM for the normalized amount of muscle Grp75 levels of untreated and LPS-treated rabbits. *Significant difference versus untreated muscles (*P *< 0.05). **(e) **to **(h) **Distribution of Grp75 immunoreactivity with indirect immunoperoxidase in hindlimb muscles of (e) untreated and (g) LPS-treated rabbits. Adjacent sections (f) and (h) allow the comparison with the distribution of type 1 fibers, identified by the immunoperoxidase staining for β-myosin heavy chain (βMy). Bar: 50 μm.

Nonregenerating muscle fibers from myositis patients apparently did not vary their Grp94 expression. We then looked for an experimental condition that would be stressful for skeletal muscle without affecting Grp94 expression in myofibers. We previously reported that systemic inflammation induced by a single LPS injection did not change Grp94 expression in rabbit adult skeletal myofibers [24] - we therefore applied the same experimental protocol to the mouse to investigate whether Grp75 and calreticulin levels were affected. A significant increase of Grp75 and calreticulin protein levels (*P *= 0.007 and *P *= 0.004, respectively) was observed in murine tibialis anterior muscles excised 48 hours after LPS administration, compared with muscles of untreated animals (Figure 5a, b). Comparable results were obtained using rabbit hindlimb muscles, excised 48 hours after a single administration of 4 mg/kg LPS [24] (Figure 5c, d). Exposure to LPS increased the intensity and distribution of Grp75 immunoreactivity within myofibers (Figure 5e, h), in the absence of histological signs of inflammation and of MHC-I upregulation (Additional file 5). A comparable, less evident pattern of reactivity was observed for calreticulin (data not shown).

At variance with LPS administration, the relative amount of Grp75 did not appear to increase in other conditions that might be accompanied by nonprimary muscle inflammation, such as the mdx strain of murine muscle dystrophy [34] (Additional file 3I). The mean ± SEM of normalized Grp75 levels of control strain C57BL/10 hindlimb muscles was 1.25 ± 0.32, whereas that for mdx muscles was 0.83 ± 0.19 (*P *= 0.26).

## Discussion

Our study shows that a large proportion of our myositis patients displayed the presence of a multiple ER stress-response in myofibers, characterized by the differential expression of the ER chaperones Grp94 and Grp75. One pattern of this ER response appeared to correlate with the degree of muscle regeneration, whereas the second pattern reflected the contribution of other influences, probably of inflammatory origin, on idiopathic myositis.

Increased expression of Grp94 and calreticulin characterized patients with a high percentage of regenerating myofibers; experimental studies showed that this ER stress-response occurred simultaneously with myotube maturation, therefore lacking specificity for myositis. Conversely, Grp75 levels increased in nonregenerating, adult, myofibers of myositis patients who were positive for sarcolemmal MHC-I immunoreactivity (Groups I and II), but also involving MHC-I-negative myofibers. Indeed, experimental animal investigations indicated that mechanisms other than MHC-I overexpression are responsible for the upregulation of Grp75, such as a systemic cytokine release.

### Increased Grp94 expression characterizes the endoplasmic reticulum stress-response that accompanies muscle regeneration

Different from most clinical studies, which consider diagnosis as the starting point, we grouped myositis patients on the presence or the absence of histological signs of inflammation and of sarcolemmal MHC-I immunoreactivity. Such an approach allowed us to circumscribe our attention to aspects, which have been related to the occurrence of an ER stress-response in muscle fibers [4].

In the present article we showed that the Grp94 level increased in biopsies that displayed more than 3% of regenerating fibers, and this association was further experimentally validated in the laboratory mouse. The crucial role played by Grp94 in muscle differentiation and maturation has been recently recognized [13-15], despite the scanty expression of this protein in adult myofibers (present manuscript and [11,24,35]). Regenerating muscle fibers recapitulated differentiation, also displaying increased immunoreactivity for calreticulin [8], another ER chaperone, whose amount decreases from early postnatal values and reduces further with aging [36], being replaced by calsequestrin [37]. In addition, regenerating myofibers showed nuclear and cytosolic staining for CHOP, whose nuclear translocation was described to occur during both *in vitro *and *in vivo *differentiation of murine myogenic cells [38,39]. The ER stress-response, which accompanies muscle differentiation, has been proposed to be responsible for the naturally occurring apoptosis during muscle development [38]. Here we have shown that the ER stress-response was present also in regenerating myofibers of patients affected with Duchenne muscular dystrophy and in those of dystrophic mdx mice, therefore reflecting the maturation of skeletal muscle cells, which occurs independently from the nature of the muscle disease.

### Inflammatory stimuli and Grp75 upregulation in myositis patients

At variance with Grp94, Grp75 protein levels increased significantly in myositis biopsies with negligible levels of regeneration, and especially in those displaying a restricted sarcolemmal MHC-I immunoreactivity to a few clusters of myofibers [40]. This observation suggested that the upregulation of this protein occurred independently from the presence of MHC-I immunoreactivity within the same fiber. In fact, Grp75 was not detectable in regenerating myofibers, despite their higher expression of MHC-I (present manuscript and [33]). Grp75 is an Hsp70 analog, mostly localized within ER and mitochondria [9]; in the present study, however, the increase in Grp75 immunoreactivity involved glycolytic fibers too, and its expression could therefore not be simply related to fiber mitochondrial content. Grp75 is, at the same time, upregulated by and protective against oxidative stress [9,18]. A potential source of oxidative stress in idiopathic myositis is represented by inflammation, through cytokines released from macrophages.

We chose LPS as the proinflammatory stimulus to experimentally reproduce systemic inflammation because it acts on Toll-like receptor 4 of skeletal muscle cells, inducing the release of chemoattractants [41], and activates NF-κB through the generation of reactive oxygen species [42]. Interestingly, a single LPS injection increased significantly Grp75 protein levels in skeletal muscle fibers, in the absence of both Grp94 [24] and MHC-I upregulation (present manuscript), proving that systemic inflammatory stimuli do raise the expression of this antioxidant stress-protein in muscle fibers. Furthermore, the lack of change in Grp75 levels in the presence of nonprimary inflammation, such as that occurring concomitantly with regeneration in the dystrophic muscle, suggests a major role of systemic versus local inflammatory stimuli in the upregulation of this stress-protein in myositis.

In addition to being upregulated by oxidative stress and involved in the antioxidant defense, increased Grp75 levels might participate in and enhance myofiber protection against complement-mediated lysis [10,43]. Although the contribution of autoantibodies in myositis remains obscure [44-46], seven out of 10 patients of the MHC-I grade + subgroup, who showed strongly increased immunoreactivity for Grp75, were positive for myositis-specific or associated autoantibodies. Until now, no convincing demonstration of sarcolemmal deposition of complement C5b-9 complex has been provided for major inflammatory muscle diseases - and neither did we succeed in detecting it. The possibility that Grp75 operates in removing this complex from the cell surface, however, makes this negative finding more likely.

### Study limitations

We are aware that the present findings suffer from limitations. Although the use of muscle biopsies from young healthy subjects as controls allowed us to exclude unwanted effects secondary to trauma or neoplastic disease (systemic inflammation) on the cell stress-response, this group differed in average age from the enrolled patients. This feature might affect the correct evaluation of the basal level of ER chaperone expression; the slightly higher values of Grp75 levels detected in Group 0 patients compared with those of Group III patients, however, would add even more relevance to the findings concerning patients of Groups I and II.

## Conclusions

Our data show that the two types of ER stress-response detectable in the presence of autoimmune myositis reveal the presence and the degree of muscle regeneration, by means of increased Grp94 levels, and reveal the influence of systemic stressing stimuli, probably of inflammatory origin, which are responsible for the increased expression of Grp75. Whereas one pattern of ER stress-response occurs independently from the pathogenesis of the disease, the increase in Grp75 levels might represent a myositis-specific feature - especially for those patients classified following Bohan and Peter's criteria and showing occasional immunoreactivity for sarcolemmal MHC-I.

## Abbreviations

ANOVA: analysis of variance; ER: endoplasmic reticulum; FITC: fluorescein isothiocyanate; LPS: lipopolysaccharide; MHC-I: class I major histocompatibility complex antigen; PBS: phosphate-buffered saline; My: myosin heavy chain; SEM: standard error of the mean.

## Competing interests

The authors declare that they have no competing interests.

## Authors' contributions

MV carried out the animal experimental studies, participated in the design of the study and drafted the manuscript. AD selected the patient cohorts, participated in the design of the study and drafted the manuscript. ET carried out immunohistochemistry and western blot analyses on patient biopsies. AG carried out the immunological profile of myositis patients. LG conceived the study, participated in its design and coordination, and helped to draft the manuscript. All authors read and approved the final manuscript.

## Supplementary Material

Additional file 1 **Therapy of patients at time of biopsy**. The table illustrates patients' individual therapy. F, female; M, male. Click here for file

Additional file 2 **Immunoreactivity for ER stress-markers in human skeletal muscle**. Serial cryosections from biopsies of a Group 0 subject (S6; A to C) were stained with indirect immunoperoxidase with antibodies for CHOP, complement 9 (C9) and ubiquitin (ubi). Bar: 100 μm. Click here for file

Additional file 3 **Immunoreactivity for ER stress-proteins and MHC-I in nonprimary inflammatory myopathies**. Serial cryosections from a Duchenne patient [31] were stained with indirect immunoperoxidase with antibodies for embryonic skeletal myosin heavy chain (εMy; A), Grp94 (B), calreticulin CRT (C), MHC-I (D), Grp75 (E). Arrows indicate regenerating myofibers positive for all markers, except Grp75. (F) to (H) Indirect immunoperoxidase labeling of tibialis anterior muscle of mdx mouse for εMy (F), Grp94 (G) and MHC-I (H) in a cluster of regenerating myofibers. Bars: 100 μm. (I) Representative western blot analysis of mdx and C57BL/10 hindlimb muscle homogenates with Grp75 and CRT. Staining of α-actinin is shown as a reference for loading. Click here for file

Additional file 4 **ER stress-response and adult myofiber necrosis**. Serial cryosections from Group I myositis Patient P2 were stained with indirect immunoperoxidase with antibodies for calreticulin CRT (A), CHOP (B) complement 9 (C9), a marker of necrosis (C) and embryonic skeletal myosin heavy chain (εMy; D). Bar: 100 μm. Click here for file

Additional file 5 **Immunoreactivity for MHC-I in animal experimental model of systemic inflammation**. Panels illustrate the representative, indirect immunoperoxidase staining of murine MHC-I in tibialis anterior cryosections of control (A) and LPS-treated (B) CD-1 mice. Only endothelial cells of capillary and small vessels appear labeled. Bar: 50 μm. Click here for file
