# Supplementary material for: Myofiber stress-response in myositis: parallel investigations on patients and experimental animal models of muscle regeneration and systemic inflammation
Source: Arthritis Res Ther. 2010 Mar 24;12(2):R52. doi: 10.1186/ar2963 (PMC2888201; doi:10.1186/ar2963)
Supplement: Additional file 1 — Therapy of patients at time of biopsy. The table illustrates patients' individual therapy. F, female; M, male. [file ar2963-S1.DOC]

| Patient  No. | Sex | Prednisone  (mg/die) | Cyclosporine  (mg/die) | Azathioprine  (mg/die) | Methotrexate  (mg/week) |
| --- | --- | --- | --- | --- | --- |
|
|
| Group I |  |  |  |  |  |
| 1 | F | 12.5 | - | - | - |
| 2 | F | - | - | - | - |
| 3 | F | 5 | - | - | 10 |
| 4 | F | 12.5 | - | - | - |
| 5 | M | - | - | - | - |
| 6 | F | 10 | - | - | - |
| 7 | F | 25 | - | - | - |
| 8 | F | 12.5 | - | - | - |
| 9 | M | - | - | - | - |
| 10 | M | 5 | - | - | - |
| 11 | F | - | - | - | - |
| 12 | F | - | - | - | - |
| 13 | M | 25 | - | - | - |
| 14 | F | 10 | 250 | - | - |
| Group II |  |  |  |  |  |
| 15 | F | - | - | - | - |
| 16 | M | - | - | - | - |
| 17 | F | 5 | - | - | - |
| 18 | M | 25 | - | - | - |
| 19 | F | - | - | - | - |
| 20 | F | 12.5 | - | - | 10 |
| 21 | F | - | - | - | - |
| Group III |  |  |  |  |  |
| 22 | F | - | - | - | - |
| 23 | F | - | - | 100 | - |
| 24 | F | 4 | - | - | - |
| 25 | M | 12.5 | - | - | - |
| 26 | F | - | - | - | - |
| 27 | F | 8 | - | - | - |
| 28 | M | - | - | - | - |
| 29 | F | - | - | - | - |
