# Supplementary figures and images for: Myofiber stress-response in myositis: parallel investigations on patients and experimental animal models of muscle regeneration and systemic inflammation
Source: Arthritis Res Ther. 2010 Mar 24;12(2):R52. doi: 10.1186/ar2963 (PMC2888201; doi:10.1186/ar2963)

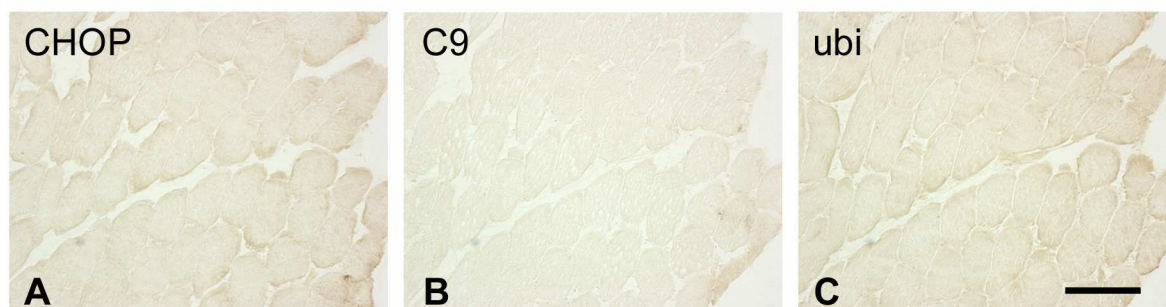

Additional File 2

Supplement: Additional file 2 — Immunoreactivity for ER stress-markers in human skeletal muscle. Serial cryosections from biopsies of a Group 0 subject (S6; A to C) were stained with indirect immunoperoxidase with antibodies for CHOP, complement 9 (C9) and ubiquitin (ubi). Bar: 100 μm. [file ar2963-S2.PDF]

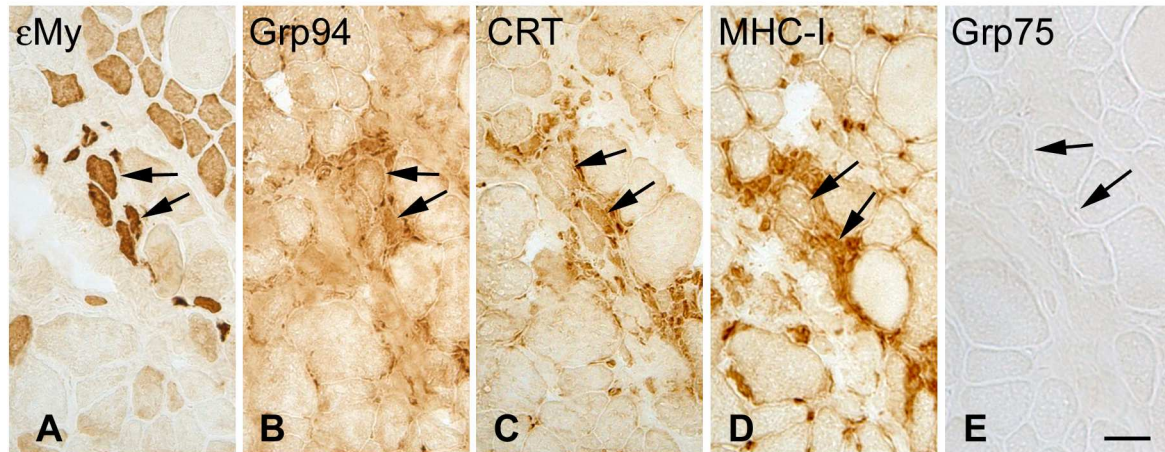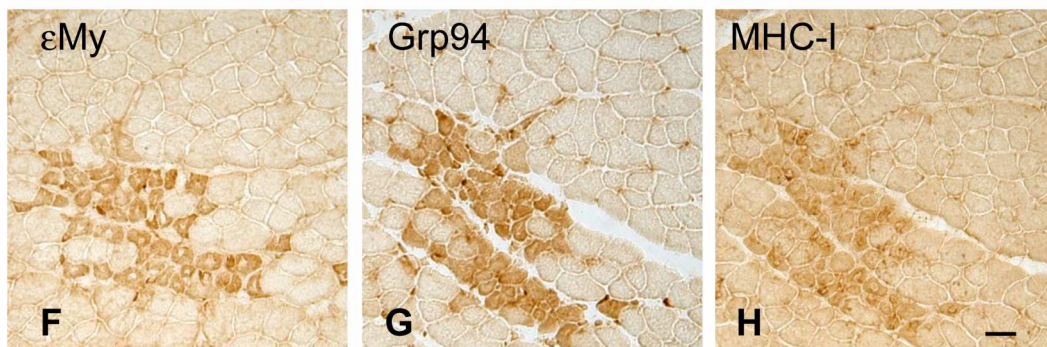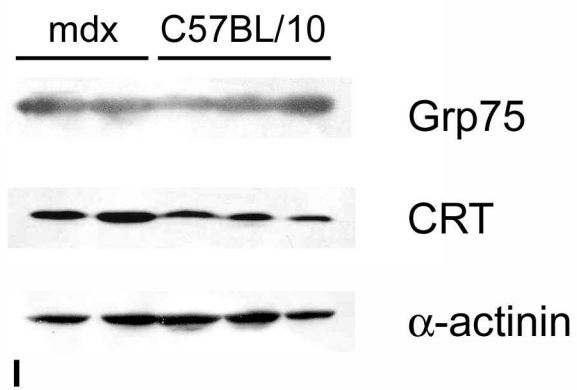

Additional File 3

Supplement: Additional file 3 — Immunoreactivity for ER stress-proteins and MHC-I in nonprimary inflammatory myopathies. Serial cryosections from a Duchenne patient [31] were stained with indirect immunoperoxidase with antibodies for embryonic skeletal myosin heavy chain (εMy; A), Grp94 (B), calreticulin CRT (C), MHC-I (D), Grp75 (E). Arrows indicate regenerating myofibers positive for all markers, except Grp75. (F) to (H) Indirect immunoperoxidase labeling of tibialis anterior muscle of mdx mouse for εMy (F), Grp94 (G) and MHC-I (H) in a cluster of regenerating myofibers. Bars: 100 μm. (I) Representative western blot analysis of mdx and C57BL/10 hindlimb muscle homogenates with Grp75 and CRT. Staining of α-actinin is shown as a reference for loading. [file ar2963-S3.PDF]

CRT

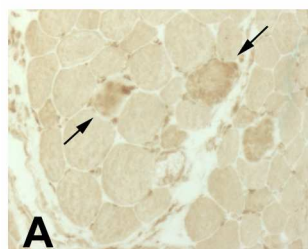

CHOP

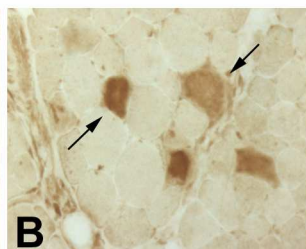

C9

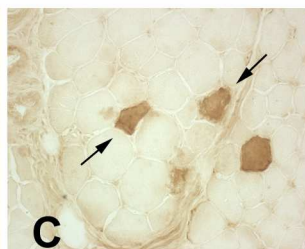

$\epsilon$ My

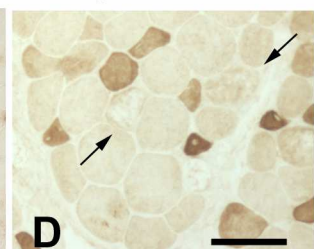

Additional File 4

Supplement: Additional file 4 — ER stress-response and adult myofiber necrosis. Serial cryosections from Group I myositis Patient P2 were stained with indirect immunoperoxidase with antibodies for calreticulin CRT (A), CHOP (B) complement 9 (C9), a marker of necrosis (C) and embryonic skeletal myosin heavy chain (εMy; D). Bar: 100 μm. [file ar2963-S4.PDF]

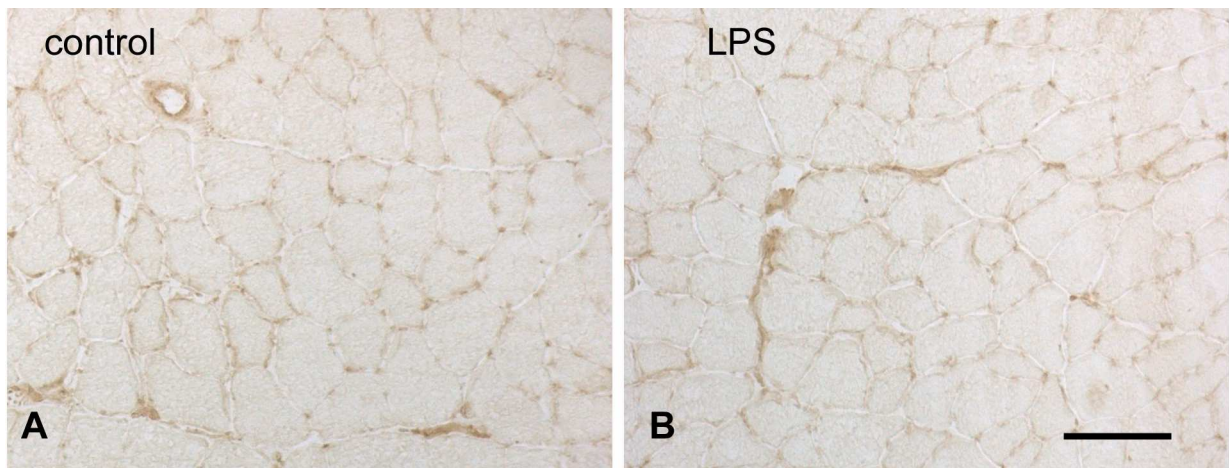

Additional File 5

Supplement: Additional file 5 — Immunoreactivity for MHC-I in animal experimental model of systemic inflammation. Panels illustrate the representative, indirect immunoperoxidase staining of murine MHC-I in tibialis anterior cryosections of control (A) and LPS-treated (B) CD-1 mice. Only endothelial cells of capillary and small vessels appear labeled. Bar: 50 μm. [file ar2963-S5.PDF]
